# Production and characterization of virus-like particles of grapevine fanleaf virus presenting L2 epitope of human papillomavirus minor capsid protein

**DOI:** 10.1186/s12896-019-0566-y

**Published:** 2019-11-21

**Authors:** Razieh Yazdani, Masoud Shams-Bakhsh, Afshin Hassani-Mehraban, Seyed Shahriar Arab, Nicolas Thelen, Marc Thiry, Jacques Crommen, Marianne Fillet, Nathalie Jacobs, Alain Brans, Anne-Catherine Servais

**Affiliations:** 1 Plant Pathology Department, Faculty of Agriculture, Tarbiat Modares University, Pajouhesh Blvd., Tehran to Karaj highway, Tehran, Iran; 2 Laboratory for the Analysis of Medicines (LAM), Department of Pharmaceutical Sciences, CIRM, University of Liège, Quartier Hôpital, B36, Tower 4, Avenue Hippocrate, 15, 4000 Liège, Belgium; 3 TAK Inspection Co, Karegar Ave, Tehran, 346 Iran; 4 Department of Biophysics, Faculty of Biological Sciences, Tarbiat Modares University, Tehran, Iran; 5 Cellular and Tissular Biology, GIGA-Neurosciences, University of Liège, Liège, Belgium; 6 Cellular and Molecular Immunology, GIGA-Research, University of Liège, Liège, Belgium; 7 Center for Protein Engineering, University of Liège, Chemistry Institute B6, 4000 Liège (Sart Tilman), Belgium

**Keywords:** Bionanoparticles, Heterologous expression systems, Protein refolding, HPV, *Nepovirus*, Vaccine antigen

## Abstract

**Background:**

Virus-like particle (VLP) platform represents a promising approach for the generation of efficient and immunogenic subunit vaccines. Here, the feasibility of using grapevine fanleaf virus (GFLV) VLPs as a new carrier for the presentation of human papillomavirus (HPV) L2 epitope was studied. To achieve this goal, a model of the HPV L2 epitope secondary structure was predicted and its insertion within 5 external loops in the GFLV capsid protein (CP) was evaluated.

**Results:**

The epitope sequence was genetically inserted in the αB-αB^”^ domain C of the GFLV CP, which was then over-expressed in *Pichia pastoris* and *Escherichia coli*. The highest expression yield was obtained in *E. coli.* Using this system, VLP formation requires a denaturation-refolding step, whereas VLPs with lower production yield were directly formed using *P. pastoris*, as confirmed by electron microscopy and immunostaining electron microscopy. Since the GFLV L2 VLPs were found to interact with the HPV L2 antibody under native conditions in capillary electrophoresis and in ELISA, it can be assumed that the inserted epitope is located at the VLP surface with its proper ternary structure.

**Conclusions:**

The results demonstrate that GFLV VLPs constitute a potential scaffold for surface display of the epitope of interest.

## Backgound

Virus-like particles (VLPs) are among the most easily-produced nanomaterials, since they result from the self-assembly of the capsid protein (CP). VLPs can be modified either genetically or chemically for various downstream applications i.e. as biomaterials [1], drug-delivery systems, bio-imaging and chemical tools [2–4], for their catalytic role [5–8] but also as vaccines [9–11]. Among the different types of vaccines, the use of subunit vaccines has been considered as a safer approach than that of inactivated pathogens [12]. However, such small molecules, e.g. composed of oligopeptides, act as weak immunogens [13]. A substantial increase in the immune response is observed when relevant epitopes are placed on the surface of macromolecular carriers, like large proteins or highly ordered structures such as VLPs [14].

Plant viruses are particularly interesting since they are not infectious for humans and animals. Compared to most animal viruses, the structure of the majority of plant viruses is very simple and often made up of a single CP type. Moreover, most of them are nonenveloped and they present a very high level of accumulation in their host [15–19]. In recent years, the potential use of plant virus-derived VLPs as novel proteic scaffold for displaying foreign epitopes has been studied. Immunogenic foreign peptides can be either genetically or chemically fused to the plant virus CP. Some of these virus platforms have been tested as potential subunit vaccines to prevent human or animal viral diseases [15, 16, 20, 21].

VLPs based on several plant viruses were tested, including cowpea mosaic virus (CPMV), alfalfa mosaic virus, tobacco mosaic virus, potato virus C, tomato bushy stunt virus, zucchini yellow mosaic virus, plum pox virus, papaya mosaic virus, cucumber mosaic virus and cowpea chlorotic mottle virus (CCMV) [22–27]. In some cases, VLP assembly takes place under certain conditions such as pH, ionic strength and presence of the viral genomic RNA. Morever, the potential insertion sites within the VLPs are usually limited based on the protein structure [28]. Therefore, there is a need for searching novel and versatile VLP systems. To this end, grapevine fanleaf virus (GFLV) has not been extensively studied as VLP-based platform for foreign epitope presentation. GFLV is a bipartite, linear, single stranded positive sense RNA genome virus which belongs to the genus *Nepovirus*. Considering the structural features of the GFLV capsid, it is composed of 60 copies of a single coat protein (504 amino acids, 56 kDa) without any envelope. The ternary structure of the CP subunit is defined by three jelly-roll domains named C, B and A from the N- to C- termini, respectively. The viral structure has been elucidated at 2.7 Å resolution [29].

Recently, GFLV VLPs have been transiently expressed using pEAQ-HT vector in *Nicotiana benthamiana* [30]. These authors showed that epitopes inserted in the N- and C-termini were exposed on the particle surface. However, no study related to insertion sites within the internal domains of GFLV VLP is available. To the best of our knowledge, the in vitro assembly of GFLV VLPs has never been studied.

VLPs from plant viruses have been produced in various microbial expression systems, namely *Escherichia coli*, *Pseudomonas fluorescens*, *Pichia pastoris* and *Saccharomyces cerevisiae* [26, 31–34]. These expression systems include prokaryotic and eukaryotic organisms, each having its own advantages and drawbacks. Indeed, production in prokaryotic systems is generally cost-effective and can take place in a short period of time. However, the lack of post-translational modifications and the presence of endotoxins are the most intriguing problems when using these systems [35]. Moreover, since large amount of proteins is often produced in an insoluble form, extra denaturation and refolding processes may be required, as it was the case for CCMV [26]. Concerning the yeast expression system, the quantity of the produced proteins is usually lower than in *E. coli*. Nevertheless, soluble proteins are obtained without an extra solubilization step. Moreover, this system is easy to manipulate and offers high expression yields as well as ease of scale-up [36].

High-risk genotypes of human papillomavirus (HPV) are responsible for cancers in females and males. Around 70 % of the cervical cancers are due to HPV16 and 18 genotypes [37]. The current vaccines are based on the major capsid protein (L1) giving rise to a HPV type-restricted protection. Instead, the HPV minor capsid protein (L2) contains cross-type neutralizing epitopes that broaden protection against various HPV types when they are displayed on a VLP platform [21]. HPV L2 epitopes (up to 36 amino acids) have been displayed on VLPs of HPV16 L1, bacteriophages PP7, MS2 and potato virus X that were expressed in baculoviruses, bacteria and plants [21, 38–40].

Here we have studied the feasibility of using GFLV VLPs as scaffold for HPV L2 epitope presentation albeit maintaining VLP formation. To achieve this goal, GFLV VLPs displaying HPV L2 epitope as well as GFLV CP VLPs were expressed in both *Escherichia coli* and *Pichia pastoris* systems.

## Results

### GFLV-L2 VLP modeling and selection of the epitope insertion and fusion site

**Fig. 1:**
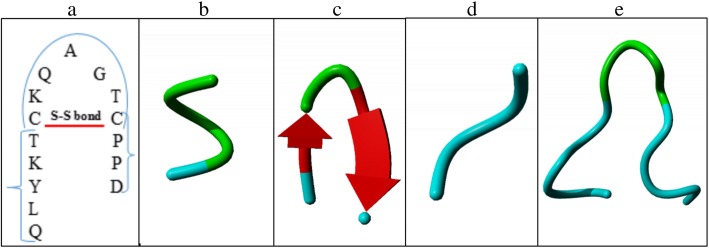
Secondary structure model of HPV L2 epitope (amino acids 17–31). **a** HPV L2 epitope sequence with the identified disulfide bond, (**b**) Structural template for the QLYKT sequence (PDB-ID: 3H9C), (**c**) Structural template for the CKQAGTC sequence (PDB-ID: 1L6P), (**d**) Structural template for the CPPD sequence (PDB-ID: 1FVU), (**e**) HPV L2 epitope secondary structure model

For GFLV-L2 VLP modeling, the secondary structures of the GFLV CP and the selected HPV L2 epitope (amino acids 17–31) were needed. The GFLV CP 3D structure was previously solved by [29] (Protein Data Bank (PDB) ID: 4V5T). In the absence of a resolved structure for the HPV L2 epitope as well as of potential structural templates in PDB using the selected sequence, the secondary structure model of the HPV L2 epitope was constructed by examining small epitope fragment secondary structures in Protein Design Assistant (ProDA) and by using information about HPV L2 structure obtained from a previous study performed by Tumban and coworkers [21]. These authors showed that the targeted HPV L2 region contains two cysteine residues (C22 and C28) which form a disulfide bond in their native conformation and both cysteine residues are conserved throughout the Papillomaviridae. We have assumed that the disulfide bond between the HPV L2 cysteine residues creates a turn structure (Fig. 1a). The HPV L2 epitope sequence was divided into three parts, namely QLYKT, CKQAGTC and CPPD, which were used as query sequences in ProDA. The structural template of the QLYKT sequence was that from amino acids 416–420 of methionyl-tRNA synthetase protein (PDB-ID: 3H9C) (Fig. 1b) that forms an α helix structure. For the CKQAGTC part, the closest structural template having a turn structure with a disulfide bond was amino acids 103–109 of disulfide bond oxidoreductase D (DsbD) protein (PDB-ID: 1L6P) (Fig. 1c). For the third part, amino acids 402–405 of botrocetin protein was used as structural template (PDB-ID: 1FVU) (Fig. 1d). Finally, the PDB files of the described structures were used as templates to model HPV L2 epitope using MODELLER software [41] (Fig. 1e).

**Fig. 2:**
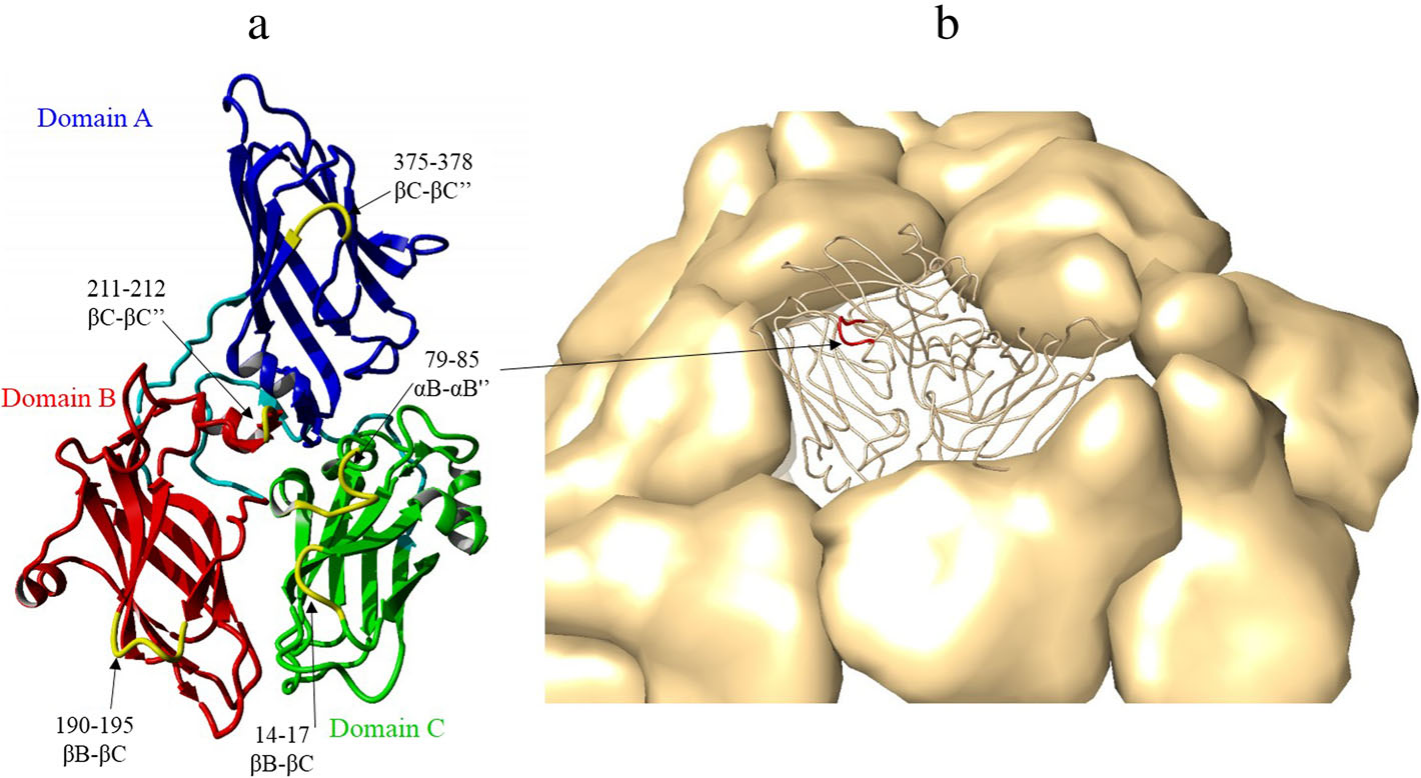
HPV L2 epitope display site on GFLV VLPs. GFLV structure was established according to data from PDB ID: 4V5T, and visualized by YASARA software (http://www.yasara.org/). **a** Ribbon diagram of the GFLV CP displaying 5 loops, 14–17 (βB-βC domain C), 79–85 (αB-αB^”^ domain C), 190–195 (βB-βC domain B), 211–212 (βC-βC^”^ domain B), 375–378 (βC-βC^”^ domain A) as potential insertion sites, (**b**) GFLV particle showing an icosahedral asymmetric unit consisting of one identical subunit. The arrow indicates the 79–85 (αB-αB^”^ domain C) external loop on GFLV CP

Based on the GFLV CP 3D structure obtained from YASARA software, 5 insertion sites for the foreign epitope were investigated, namely amino acid residues 14–17 (βB-βC domain C), 79–85 (αB-αB^”^ domain C), 190–195 (βB-βC domain B), 211–212 (βC-βC^”^ domain B), 375–378 (βC-βC^”^ domain A). These sites were assumed to have at least one advantage for the insertion of foreign epitopes, i.e. the fact that the inserted epitope would be displayed on the surface of the particle since these regions are located in the outer area of the particle (Fig. 2a); The 190–195, 211–212 and 375–378 loops were found to be unsuitable as insertion regions. Indeed, they are located between two sheets and deletion/insertion or replacement in these loops would probably disturb β-barrel structures and have a critical effect on the conformational structure. Two insertion sites, amino acids 79–85 and 14–17, would have most likely a minimal impact on the particle formation, meaning that an insertion in these loops would not interfere in protein-protein interactions. Finally, the 79–85 loop was selected for the insertion of the HPV L2 epitope on the GFLV VLP surface. The distance between both ends of the 79–85 loop (about 10 Å) was similar to that between both ends of the HPV L2 epitope. It was assumed that the HPV L2 epitope would have a better conformational flexibility in the 79–85 loop than in the 14–17 loop and that a disulfide bond will be formed in this loop (Fig. 2b) (detailed information is not shown).

### Cloning, expression and production of GFLV VLPs in *E. coli*

Restriction enzyme analysis confirmed that the GFLV CP and GFLV L2 genes (1.5 kb) were inserted in the pET26B vector between *Nco*I-*Bam*HI cloning sites. Further, nucleotide sequencing using specific primers confirmed the insertion of the GFLV CP and GFLV L2 genes in frame with pelB leader sequence without any mutation. Additional file 1: Figure S1A shows a SDS-PAGE analysis of protein expression in *E. coli* strains BL21 (DE3). The detection of a distinct band at 56–57 kDa in lanes 3 and 4 indicates that large amounts of the recombinant GFLV CP and GFLV L2 in an insoluble form were produced in *E. coli*.

Various expression conditions were examined in order to obtain the GFLV CP and GFLV L2 in a soluble form in *E. coli*, such as different *E. coli* strains (BL21 and SHuffle T7 cells), induction times (2, 4, 6 and 8 h), isopropyl β-D-1-thiogalactopyranoside (IPTG) concentrations (0.25, 0.5 and 0.8 mM) and growth temperatures (15, 25 and 37 °C). However, under all tested conditions, the proteins were expressed as inclusion bodies (IBs). The highest expression yield was obtained using the strain BL21 (DE3), 0.25 mM IPTG at 37 °C during 4 h in a shaker.

To solubilize the proteins of interest, different solutions containing one or several compounds were tested (8 M urea and 1% triton X-100; 8 M urea, 1% triton X-100 and 10 mM dithiothreitol (DTT); 8 M urea, 1% triton X-100, 10 mM DTT and 14 mM mercaptoethanol, pH = 8 for 4 and 6 h at 4 °C; 10 mM SDS and 15 mM DTT; 8 M urea and 10 mM DTT, pH = 7 for 16 h at 4 °C). The IBs were found to be solubilized in solutions containing 10 mM SDS and 15 mM DTT or 8 M urea and 10 mM DTT, pH = 7 for 16 h at 4 °C. The solubilization of the IBs in the solution containing urea during 16 h at 4 °C was found to be the best protocol. Indeed, as shown in Additional file 1: Figure S1B, the GFLV CP and GFLV L2 were almost completely solubilized under these conditions. The proteins were then purified by size exclusion chromatography (SEC) using a Superdex 200 column. For the VLP assembly from the purified GFLV CP and GFLV L2, different buffers were evaluated. It was found that an acidic pH and a high ionic strength led to protein aggregation at both temperatures (4 and 25 °C). The GFLV CP and GFLV L2 were found to self-assemble into VLPs after two steps of dialysis in HEPES buffer at pH 8 and 4 °C. GFLV VLPs were then concentrated by sucrose cushion ultracentrifugation. As can be seen in Additional file 1: Figure S1C, highly purified GFLV CP and GFLV L2 VLPs could be obtained (estimated purity: 94 and 93%, respectively).

### Cloning, expression and production of GFLV VLPs in *P. pastoris*

The correct sequences and frames for sequential translation of the α-factor and GFLV CP or GFLV L2 fragments into pPICZα were confirmed by sequencing. GFLV CP and GFLV L2 were expressed after three days of culture and in the presence of 2% methanol. They were secreted into the culture medium along with the α-factor signal which was cleaved by the Kex2 protease. The supernatants from the positive colonies that grew in the highest concentration of Zeocin™ were analyzed by SDS-PAGE after concentration by ultracentrifugation (cf. Additional file 2: Figure S2A). As shown in this Figure, A1 clone of GFLV CP and A3 clone of GFLV L2 (lanes 1 and 3) were found to secrete high protein levels while the GFLV CP and GFLV L2 were not expressed in A2 and A4 clones, respectively (lanes 2 and 4). The proteins of interest were not observed in the culture of *P. pastoris* transformed with the empty pPICZα vector in the presence of methanol (lane 5). The cultures of the selected clones were concentrated by sucrose cushion ultracentrifugation (cf. Additional file 2: Figure S2B; estimated purity: 97% for GFLV CP VLPs and 98% for GFLV L2 VLPs).

### Characterization of the capsid proteins

**Fig. 3:**
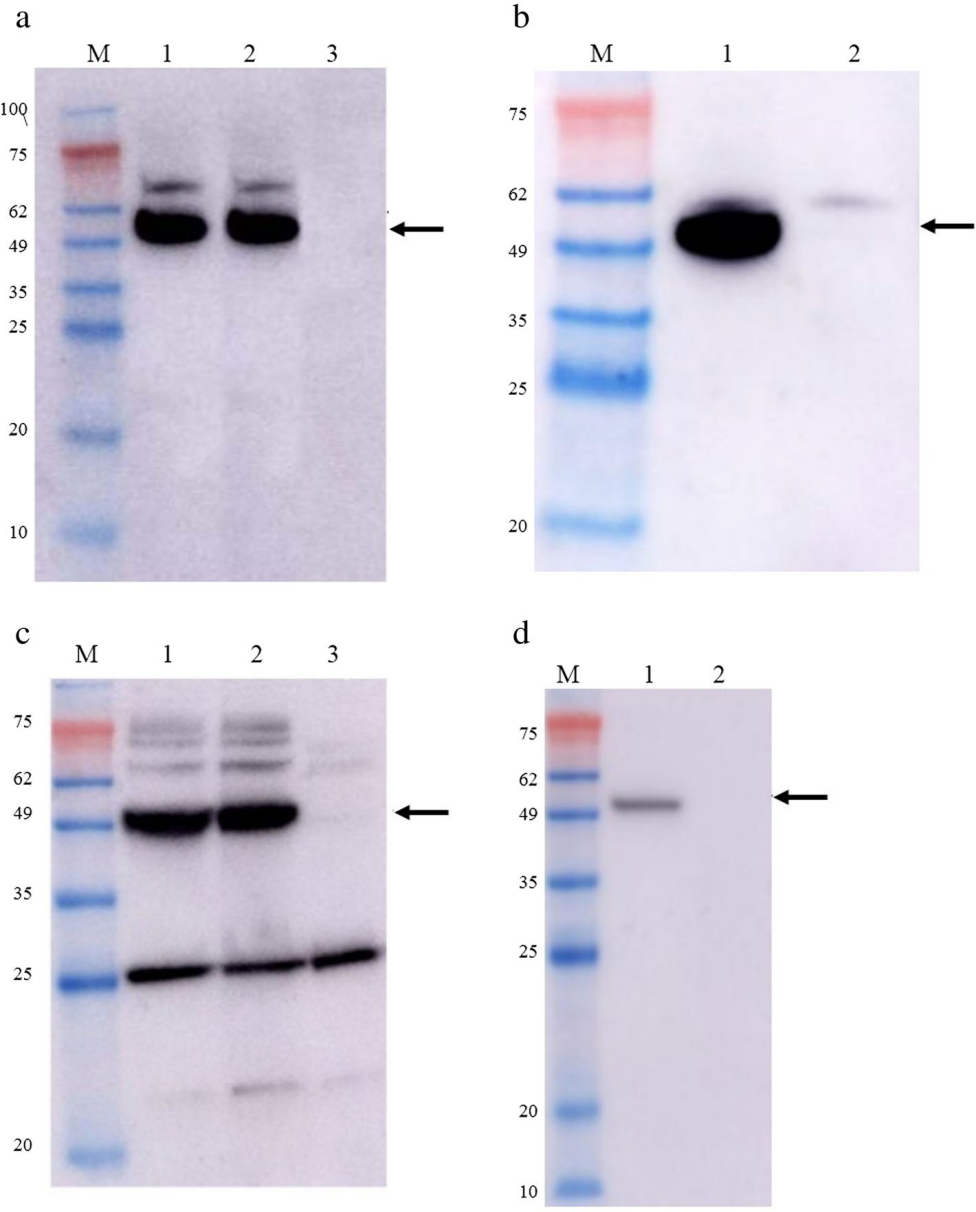
Western blot analysis of the GFLV CP and GFLV L2 VLPs expressed *in E. coli* (**a** and **b**) and in *P. pastoris* (**c** and **d**). **a** and **c**: blots were probed with anti-GFLV CP polyclonal antibody. Lane M: standard protein molecular weight markers, lane 1: GFLV CP, lane 2: GFLV L2, lane 3: negative control (*E. coli* with pET26 empty vector or *P. pastoris* with pPICZα empty vector). **b** and **d**: blots were probed with mouse anti-HPV L2 antibody. Lane M: standard protein molecular weight markers, lane 1: GFLV L2, lane 2: GFLV CP

Western blot analysis using an antibody directed against the GFLV CP showed the presence of the protein of interest for the GFLV L2 samples (cf. Fig. 3a (*E. coli*) and c (*P. pastoris*), lane 2), indicating that the GFLV antibody interacts with the GFLV L2 protein, as is the case for the GFLV CP (cf. Fig. 3a and c, lane 1). Immunoblotting directed against the HPV L2 epitope confirmed its presence in the GFLV-L2 protein (cf. Fig. 3b (*E. coli*) and d (*P. pastoris*), lane 1). Considering the absence of a band at the corresponding MW for the GFLV sample (cf. Fig. 3b (*E. coli*) and d (*P. pastoris*), lane 2), it can be assumed that the GFLV CP does not interact with the HPV L2 antibody, which therefore confirms that this antibody specifically detects the HPV L2 epitope in the GFLV L2 protein.

### Characterization of the GFLV VLPs

**Fig. 4:**
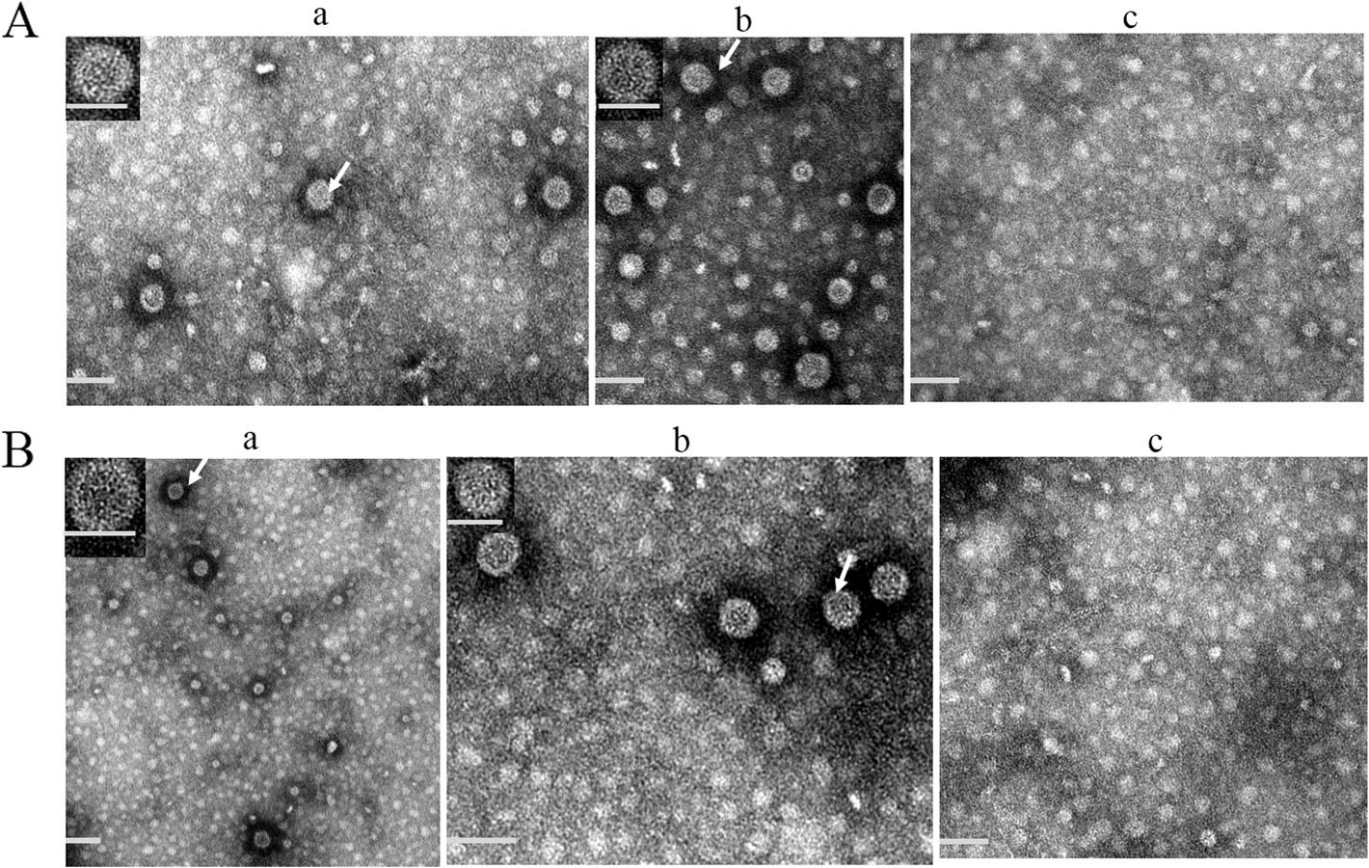
Ultrastructural analysis of VLPs obtained from *E. coli* (**A**) and from *P. pastoris* (**B**), a GFLV CP VLPs, b GFLV L2 VLPs, c negative control (*E. coli* with pET26 empty vector or *P. pastoris* with pPICZα empty vector). Scale bar: 50 nm. Arrows indicate viral particles visualized at high magnification in the inserts (scale bar in the inserts: 30 nm)

**Fig. 5:**
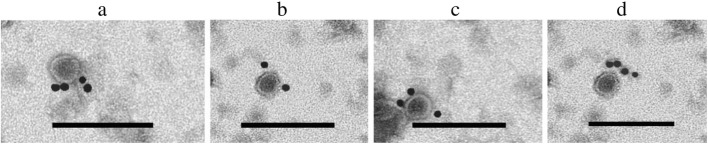
Immuno-gold labeling analysis of chimeric VLP expressed in *E. coli* and *P. pastoris*. Anti-GFLV CP polyclonal antibody is used as primary antibody. **a** GFLV CP VLPs from *E. coli*, (**b**) GFLV L2 VLPs from *E. coli*, (**c**) GFLV CP VLPs from *P. pastoris*, (**d**) GFLV L2 VLPs from *P. pastoris*. Scale bar: 100 nm

GFLV VLP pellets from both expression systems were resuspended in the virus buffer (0.1 M HEPES, 0.001 M EDTA, pH 8). VLPs were analyzed by SEC and a major peak was observed at 1.3 mL in both cases (Additional file 3: Figure S3). The final yield of VLPs from *E. coli* was 15–20 mg/L while that from *P*. *pastoris* was 1–3 mg/L. Transmission electron microscopy (TEM) analysis showed a population of spheroid particles having a diameter of 29 ± 1.5 nm and 31.9 ± 2.5 nm in *E. coli* (Fig. 4A, a and b) and *P. pastoris* (Fig. 4B, a and b), respectively. This indicates that the GFLV CP and GFLV L2 are capable of self-assembly into VLPs in vitro (*E. coli*) and in vivo (*P. pastoris*). In agreement with the TEM results, the immunostaining electron microscopy (ISEM) analysis confirmed the presence of GFLV VLPs obtained from *E. coli* and *P. pastoris* that clearly immunoreact with anti-GFLV CP polyclonal antibody (Fig. 5a-d). It should be noted that when polyclonal sera from mice immunized with HPV16 L2 peptide 1–88 were used as primary antibody, the staining was too weak to draw a conclusion about the interaction between GFLV L2 VLPs and HPV L2 antibody.

**Fig. 6:**
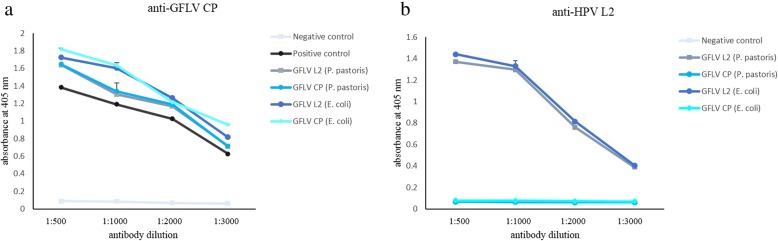
Characterization of GFLV CP and GFLV L2 VLPs by ELISA. Serial dilutions of (**a**) anti-GFLV CP and (**b**) anti-HPV L2 were tested in triplicates against GFLV CP and GFLV L2 VLP antigens. Leaf tissue infected by GFLV was used as positive control. The negative control was *E. coli* with pET26 empty vector

Both GFLV VLPs were evaluated in ELISA using anti-GFLV CP and an antibody directed against amino acids 1–40 of HPV16 L2 (cf. Fig. 6). ELISA analysis using anti-GFLV CP confirmed the formation of GFLV VLPs in *P. pastoris* and *E. coli* systems. Moreover, the insertion of HPV L2 epitope did not interfere with VLP formation. ELISA analysis using anti-HPV L2 antibody demonstrated the interaction of the antibody with GFLV L2 VLPs, indicating that the HPV L2 epitope is located on the VLP surface.

**Fig. 7:**
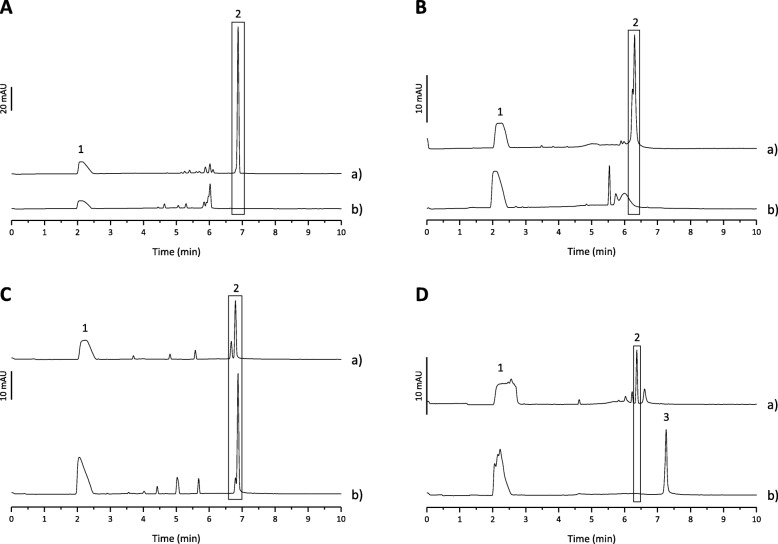
CE analysis of complex formation between GFLV CP VLPs (**A)** or GFLV L2 VLPs (**B**) and GFLV antibody. **a** VLPs analysis without antibody, **b** VLPs incubated with 200 fold molar excess of GFLV antibody during one hour at room temperature before injection. CE analysis of complex formation between GFLV CP VLPs (**C**) or GFLV L2 VLPs (**D**) and HPV L2 antibody. **a** VLPs analysis without antibody, **b** VLPs incubated with 39 fold molar excess of HPV L2 antibody during one hour at room temperature before injection. Peak 1, IS; peak 2, VLP; peak 3, GFLV L2 VLPs / HPV L2 antibody complex; BGE, 0.01 M Tris HCl, 0.01 M HEPES-Na, 0.1 M NaCl and 0.1% PEG 6000, pH 7.4 buffer containing 0.2% Tween 20 and 1.5 mM SDS; 48.5 cm (8.5 cm to the detector) × 50 μm PEO-coated capillary; voltage + 10 kV; pressure injection for 15 s at − 50 mbar; T = 15 °C; Detection at 280 nm

Capillary electrophoresis (CE) is a very powerful technique that allows the analysis of intact viral particles but also the study of biomolecular interactions under native conditions [42]. It is worth noting that the interaction of a viral particle with an antibody may lead to the appearance of a peak corresponding to the virus-antibody complex. However, if the binding of the antibody with the virus results in the aggregation of several particles, the virus-antibody complex may not be detected [43]. GFLV CP and GFLV L2 VLPs were first incubated during 1 h at room temperature with the GFLV antibody and the mixtures were then analyzed by CE (cf. Fig. 7A and B). As shown in these figures, both VLP samples were found to interact with the GFLV antibody as the VLP peak disappeared in the presence of the antibody. The CE experiments were then performed using the antibody directed against amino acids 1–40 of HPV16 L2 (cf. Fig. 7C and D). GFLV CP and GFLV L2 VLPs were also incubated during 1 h with the antibody before the CE analysis. As expected, GFLV CP VLPs did not interact with the HPV L2 antibody (cf. Fig. 7C). Indeed, following the incubation with this antibody, the VLP peak was still present and the ratios of the corrected area to that of the internal standard were the same under both conditions. Regarding the GFLV L2 sample, a complex was formed when the VLPs were incubated with the HPV L2 antibody since a peak with a longer migration time appeared (cf. Fig. 7D).

### GFLV VLPs are nucleic acid-free

The agarose gel electrophoresis of the RT-PCR products of GFLV CP and GFLV L2 mRNA that was amplified using one set of specific primers showed that CP mRNA was not encapsidated into VLPs produced from *E. coli* (lanes 1–2, Additional file 4: Figure S4) and from *P. pastoris* (lanes 3–4, Additional file 4: Figure S4). According to these results, it can be concluded that GFLV VLPs assemble independently from RNA.

## Discussion

The discovery of a rational VLP system for displaying epitopes of interest is obviously a challenging effort. Indeed, the scaffolding protein has to present a potential flexibility for any changes and the functionality of the epitope has to be preserved. Here we present a VLP platform based on GFLV particles to expose HPV L2 epitope on its surface without interfering in VLP formation.

Based on in silico data, loop structures βB-βC and αB-αB” (domain C) were predicted as epitope insertion sites and the latter was selected for expression experiments. Expression of GFLV constructs in *E. coli* ended up in the formation of IBs. This was already reported for other plant VLP systems like CCMV and Faba bean necrotic yellows virus [26, 44]. This might be due to the presence of intra- and inter-molecular disufide bonds in cysteine-rich proteins, as is the case for GFLV CP [45]. To solubilize GFLV expressed in *E. coli*, an extra step was developed. This step is required for VLP formation, as also shown for CCMV VLPs [26]. TEM examination of GFLV L2 VLPs showed the presence of spherical particles similar to GFLV particles indicating that the insertion of the HPV L2 sequence does not abolish VLP formation. In addition, the expression of the GFLV CP and GFLV L2 within *P. pastoris*, ended up with the formation of VLPs, as already reported for CCMV [33]. When comparing both expression systems used in this study, it can be concluded that in *E. coli*, the use of a fast and easy process results in a higher amount of the protein than in *P. pastoris*. However, in yeast, soluble proteins are obtained without an extra solubilization step. GFLV particles can also be produced in *N. benthamiana* but it is worth noting that the amount of protein is very low (386 to 445 μg/kg fresh leaf) [30]. Transient expression has also been used for the production of other plant virus-derived VLPs, such as CPMV [46], turnip crinkle virus [47] and *Ageratum* yellow vein virus [48]. Unlike bacterial expression systems, plants are able to provide post-translational maturations such as glycosylation and also reduce the risk of endotoxin contamination [49].

From the GFLV CP 3D structure, it could be deduced that the selected 79–85 loop of all sixty CP subunits is surface accessible. The insertion in this loop did not interfere in the CP- CP interactions necessary for VLP assembly and stability and resulted in the exposure of the HPV L2 epitope at the particle outer surface in its native conformation. In agreement with our results, Schellenberger et al. reported that the replacement in the CP region R1 of GFLV (amino acids 79–85) to CP region R1 of Arabis mosaic virus, which is located at the external surface of the particles, did not interfere with virus encapsidation [50].

Since GFLV L2 VLPs were found to interact with the HPV L2 antibody under native conditions in capillary electrophoresis and in ELISA, it can be assumed that the inserted epitope is located at the VLP surface with its proper ternary structure. This is obviously crucial for the use of GFLV L2 VLPs as a potential vaccine. Previous studies reported that this HPV L2 epitope located at the N-terminal part of MS2 phage has more conformational flexibility than in the AB-loop of PP7 phage, allowing the formation of a disulfide bond in the epitope and leading to a better presentation of the peptide, inducing a more broadly reactive antibody response against the HPV L2 epitope [21, 51].

The absence of GFLV mRNA was confirmed using purified VLPs as input for RT-PCR. However, the encapsulation of non-specific mRNA and DNA within the VLPs cannot be excluded. To avoid any encapsulation of nucleic acids, the subunits might be treated using RNaseA and DNase before VLP formation.

## Conclusions

In this study, the generation of a new icosahedral VLP carrier through the cloning and expression of the GFLV CP and GFLV L2 genes in *E. coli* and *P. pastoris* was demonstrated. VLPs containing an epitope from the HPV minor CP (HPV L2 epitope) were obtained. The application of GFLV L2 VLPs as a promising vaccine antigen will be clear after immunization tests. Besides loop αB-αB^”^, βB-βC loop might be investigated as another insertion site for possible divalent exposure of candidate peptides at the same time.

## Methods

### Prediction of HPV L2 epitope structure and insertion sites in GFLV VLP

Since the crystal structure of HPV L2 minor protein is not available, the secondary structure model of the HPV L2 epitope (*NH*_*3*_-QLYKTCKQAGTCPPD-*COOH*) was constructed by examining small epitope fragments in Protein Design Assistant (ProDA; http://bioinf.modares.ac.ir/software/linda/). To this end, the HPV L2 epitope sequence was divided into three parts, namely QLYKT, CKQAGTC and CPPD, which were used as query sequences in ProDA. Finally, the PDB files of the described structures were used as templates to model HPV L2 epitope using MODELLER software [41]. The model was visualized with YASARA software (http://www.yasara.org/).

The GFLV CP PDB code [29] was used as input to search for potential insertion sites using YASARA software**.**

### Construction and expression of GFLV CP and GFLV L2 in *E. coli*

To produce pBluescript II SK-GFLV-L2 construct, GFLV L2 gene was synthesized and cloned into the *NcoI* and *BamHI* sites by BioBasic Company (Toronto, Canada). pGEM-GFLV CP [52] and pBluescript II SK-GFLV-L2 were used as templates for amplification of GFLV CP and GFLV L2 genes using a primer set (Sense primer:5′-CCA GCC GGC GAT GGC CAT GGG ATT AGC TGG TAG AGG-3′; antisense primer: 5′-ACG GAG CTC GAA TTC GGA TCC CTA GAC TGG GAA ACT GGT TCT CCA-3′ which includes two homologous region CCA GCC GGC GAT GGC and ACG GAG CTC GAA TTC GGA TCC) and Phusion High Fidelity DNA polymerase (New England Biolabs, Ipswich, MA, USA). The amplified fragments were subcloned in the pET26 vector in frame with the pelB leader sequence using the Gibson assembling cloning master mix for 15 min at 50 °C (New England Biolabs) and transformed into *E. coli* DG1 (Eurogentec, Liege, Belgium). The grown colonies were tested and analysed using colony PCR, digestion analyses and sequencing to confirm GFLV CP and GFLV L2 constructs. Finally, the positive plasmids were transformed into *E. coli* BL21 (DE3) (New England Biolabs) using the calcium chloride transformation method.

*E. coli* strains BL21 (DE3) transformed with pET26 GFLV CP and pET26 GFLV L2 were grown overnight in Luria–Bertani (LB) medium containing 50 mg/L kanamycin. One mL of the overnight culture was added to 100 mL of fresh LB culture medium and incubated at 37 °C. When the OD600 reached a value of 0.8–1, 0.1–0.25 mM IPTG was added and the cultures were incubated at 18–37 °C for 1–4 h. Pelleted cells were collected by centrifugation and stored at − 20 °C for use in subsequent steps. The negative control was empty pET26 vector.

### In vitro denaturing and refolding of GFLV VLPs produced by *E. coli*

To solubilize GFLV CP and GFLV L2 proteins, a process of denaturing and refolding was adapted from a previous work [26] with some modifications. The insoluble proteins produced in *E. coli* were dissolved in 15 mL of disassembly buffer (0.02 M Tris-HCl, 0.01 M DTT and 8 M urea, pH 7) with slow rotation during 16 h at 4 °C. The dissolved proteins were centrifuged at 16000 rpm for 15 min. The supernatants were filtered and purified by SEC using a Superdex 200 column. SEC runs were performed at a constant flow rate of 2 mL/min using the disassembly buffer (0.02 M Tris-HCl, 0.01 M DTT, 8 M urea, pH 7) containing 2.5 M NaCl as eluent. Twenty μL of the peak fractions were analysed by SDS-PAGE. The fractions containing the purified GFLV CP and GFLV L2 were dialyzed 6 times against the dialysis buffer (0.02 M Tris-HCl, pH 7) and were refolded using 2 cycles of dialysis against the reassembly buffer (0.1 M HEPES, pH 8). Finally, the GFLV VLPs concentrated using ultracentrifugation were dissolved in the virus buffer (0.1 M HEPES, 0.001 M EDTA, pH 8) (Merck, Darmstadt, Germany).

### Construction of recombinant *P. pastoris*

The PCR of the GFLV CP and GFLV L2 genes were done using a primer set (sense primer: 5′- AAG AAG GGG TAT CTC TCG AGA AAA GAG AGG CTG AAG CTA TGG GAT TAG CTG GTA GAG GAG − 3′; antisense primer:5′- GCT GGC GGC CGC CGC CTA GAC TGG GAA ACT GGT TCT − 3′ which includes two homologous regions AAG AAG GGG TAT CTC TCG AGA AAA GAG AGG CTG AAG CTA and GCT GGC GGC CGC CGC, respectively) and the recombinant plasmid (pPICZα-CP) with α-factor sequence was constructed by Gibson assembly method [53]. Five-ten μg of pPICZα-CP DNA plasmid were linearized using PmeI enzyme and introduced in *P. pastoris* X-33 by electroporation as described in the *Pichia* Expression Kit protocol (Invitrogen, Carlsbad, CA, USA).

### Selection of multiple copy recombinant genes in *P. pastoris*

The PCR-positive colonies on YPDS-Zeocin™ (100 mg/mL) agar plates were cultured in 250 μL YPD medium and incubated for 48 h at 28–30 °C. Ten μL of each culture was then recultured in 240 μL fresh YPD medium and incubated at 30 °C for 24 h. This step was repeated 3 times. Ten μL of the last microplate was cultured in 240 μL fresh YPD medium with three concentrations of Zeocin™, i. e. 0.25 mg/mL, 0.5 mg/mL and 1.0 mg/mL. The microplates were incubated for 3–4 days at 30 °C until the cells grew.

### Laboratory scale yeast cell density and total protein expression

Sixty μL of the selected transformants were inoculated into 5 mL YPD medium and shaked at 200 rpm for 16–18 h at 30 °C. Afterwards, 20 mL of BMGY medium were inoculated with 250 μL of YPD culture and incubated as described above. When the OD600 had a value of 2–6, a centrifugation at 4800 rpm for 10 min was performed. Pelleted cells were resuspended in 20 mL of BMMY medium and incubated at 28 °C until the OD600 reached a value of 2–6. Sterile pure methanol (final concentration of 2%) was then added every 24 h to maintain induction. Every 12 h, 1 mL of the induced culture was sampled and 1 mL of BMMY medium was added to keep the original volume of the culture, for the determination of the protein expression level in the supernatant by SDS-PAGE and Western blotting. All materials used in the culture media were purchased from Sigma Aldrich (Saint-Louis, MO, USA).

### Purification and concentration of GFLV VLPs

The cultures of *P. pastoris* containing the expressed protein were first centrifuged (10,000 g) for 30 min. The supernatants were centrifuged at 73,360 g (Beckman Type 30 rotor, Brea, CA, USA) for 2 h to pellet VLPs [33]. VLPs were resuspended in the virus buffer and further purified by the sucrose cushion centrifugation. For this purpose, 2 mL of the VLP suspensions were added to 15 mL of 20% sucrose solution (w/v) and centrifuged at 40,000 rpm for 3 h. VLP pellets were again resuspended in the virus buffer and dialyzed against the virus buffer. For the concentration of the GFLV VLPs produced in *E. coli,* the sucrose cushion method described above was also applied.

### SDS-PAGE and Western blotting

The proteins expressed in both systems were analyzed in a 12% SDS-PAGE gel followed by Western blot (Bio-Rad, Hercules, CA, USA). Western blotting analysis was performed using anti-GFLV CP polyclonal antibody (1:1000) (DSMZ, Braunschweig, Germany) and mouse anti-L2 polyclonal sera (kindly provided by Dr. Ebenezer Tumban, Department of Molecular Genetics and Microbiology, University of New Mexico School of Medicine). The development of the signals was carried out using the Super Signal West Pico Chemiluminescent Substrate (Thermo Scientific, Waltham, MA, USA) (Amersham™ Imager 600).

### Transmission electron microscopy (TEM) and immunostaining electron microscopy (ISEM)

GFLV VLPs were analyzed by TEM (Jeol JEM-1400, Jeol, Zaventem, Belgium). For this purpose, 10 μL of the samples, previously diluted 10–15 times in the virus buffer were placed on 400 mesh copper grids (Laborimpex, Brussels, Belgium) for 2 min followed by negative staining. The mean particle diameter was determined on the basis of the analysis of 20 particles. The ISEM was performed following the procedure published by [54]. Thirty μL of the samples were placed on 200 mesh Formvar/Carbon Nickel grids (Laborimpex) for 60 min, followed by blocking using PBS buffer containing 1% bovine serum albumin (BSA) for 20 min. Anti-GFLV CP polyclonal antibody (1:100) or anti-L2 polyclonal sera (kindly provided by Dr. Ebenezer Tumban) was used as primary antibody and goat anti-rabbit-gold (10 nm) conjugates (AURION, Wageningen, Netherlands) were employed as secondary antibody at a 1:40 dilution in PBS buffer for 60 min (supplemented with 0.2% BSA and normal goat serum, 1:50). After 5 washing steps, samples were postfixed for 10 min in 2.5% glutaraldehyde and counterstained using 2.5% uranyl acetate for 10 min followed by 4 washes and an incubation of 10 min in lead citrate. Grids were finally washed 4 times in deionized water and examined by TEM (Jeol JEM-1400) at 80 kV using GFLV virion derived VLPs as a positive control. When the secondary antibody was omitted, no label occurred (data not shown).

### ELISA

To confirm VLP formation and to evaluate the exposition of HPV L2 epitope on the VLP surface, ELISA analysis was performed as previously described in [55, 56]. VLP antigens were diluted in 0.1 M PBS (pH 7.2; final concentration: 1000 ng/mL) and kept on ice prior to the analysis. VLPs were tested using serial dilutions of rabbit anti-GFLV polyclonal antibody (1:500, 1:1000, 1:2000, 1:3000) (DSMZ) and mouse monoclonal antibody raised against amino acids 1–40 of HPV16 L2 (1:500, 1:1000, 1:2000, 1:3000) (Santa Cruz, Heidelberg, Germany). Goat anti-rabbit-IgG and goat anti-mouse-IgG alkaline phosphatase conjugates (1:3000) (Promega, USA) were used as secondary antibodies. The wells were developed in 4-nitrophenyl phosphate disodium salt hexahydrate (Sigma-Aldrich) and the absorbance was measured using an ELISA reader Anthos 2020 at 405 nm.

### Size exclusion chromatography (SEC)

VLPs were purified by size exclusion chromatography on a 2 mL column of Sephacryl 300 using 100 mM HEPES and 0.001 M EDTA (pH 8) buffer.

### Capillary electrophoresis (CE)

The experiments were performed on a HP^3D^ CE system (Agilent Technologies Waldbronn, Germany). This instrument is equipped with an autosampler and a temperature control system (15–60 °C ± 0.1 °C). The detection was carried out using an on-column diode array detector. UV detection was set at 280 nm. Bare fused-silica capillaries with an internal diameter of 50 μm were purchased from Optronis (Kehl, Germany). Capillaries of 48.5 cm total length (8.5 cm effective length) were dynamically coated with poly (ethylene oxide) (PEO) according to the procedure previously reported by [57]. Experiments were performed using the outlet injection mode. VLP samples were injected hydrodynamically by applying a pressure of − 50 mbar during 15 s. Anti-GFLV antibody (DSMZ) and mouse monoclonal antibody raised against amino acids 1–40 of HPV16 L2 (Santa Cruz) were used for affinity experiments. The separation was performed applying a voltage of 10 kV (normal polarity mode) and the capillary was thermostated at a temperature of 15 °C.

### Detection of nucleic acid in GFLV VLPs

RT-PCR test was performed to evaluate the presence or the absence of nucleic acid in GFLV VLPs. Total RNA was purified from VLPs using Trizol according to the manufacturer’s instructions (Invitrogen, Carlsbad, CA, USA) and specific primers of GFLV CP were used for the detection of viral mRNA in the VLPs.

## Supplementary information

**Additional file 1: Figure S1.** (**A**) SDS–PAGE analysis of the protein expression in *E. coli*. Lane M: unstained PageRuler® molecular weight markers, lane 1: Total fraction of GFLV-L2, lane 2: soluble fraction of GFLV-L2, lane 3: insoluble fraction of GFLV-L2, lane 4: insoluble fraction of GFLV CP. (**B**) SDS–PAGE analysis after application of the solubilisation protocol. Lane M: unstained PageRuler® molecular weight markers, lane 1: pellet fraction of GFLV CP, lane 2: pellet fraction of GFLV L2, lane 3: supernatant fraction of GFLV CP, lane 4: supernatant fraction of GFLV L2. (**C**) SDS–PAGE analysis after sucrose cushion ultracentrifugation. Lane M: unstained PageRuler® molecular weight markers, lane 1: GFLV CP VLPs, lane 2: GFLV L2 VLPs. The proteins of interest are indicated by an arrow. Gels were stained with coomassie blue.

**Additional file 2: Figure S2.** (**A**) SDS-PAGE analysis of the GFLV VLP samples expressed in *P. pastoris*. Lane M: unstained PageRuler® molecular weight markers, lane 1: clone A1 of GFLV CP, lane 2: clone A2 of GFLV CP, lane 3: clone A3 of GFLV L2, lane 4: clone A4 of GFLV L2, lane 5: negative control (*P. pastoris* with pPICZα empty vector). (**B**) SDS–PAGE analysis of the GFLV VLP samples expressed in *P. pastoris* after sucrose cushion ultracentrifugation. Lane M: unstained PageRuler® molecular weight markers, lane 1: clone A1 of GFLV CP, lane 2: clone A3 of GFLV L2. Gel was stained with coomassie blue.

**Additional file 3: Figure S3.** Size exclusion chromatography (SEC) analysis of GFLV VLPs using a Sephacryl 300 column (absorbance at 280 nm). (**A)** SEC analysis of the VLPs produced in *E. coli*, **a** GFLV CP VLPs, **b** GFLV L2 VLPs. (**B)** SEC analysis of the VLPs produced in *P. pastoris,* **a** GFLV CP VLPs, **b** GFLV L2 VLPs.

**Additional file 4: Figure S4.** Analysis of RT-PCR products of GFLV CP and GFLV L2 mRNA by agarose gel electrophoresis. Lanes 1–2: nucleic acids extracted from 200 μL GFLV CP and GFLV L2 VLPs samples from *E. coli*, lanes 3–4: nucleic acids extracted from 200 μL GFLV CP and GFLV L2 VLPs samples from *P. pastoris.* Lane 5: positive control (i.e. nucleic acids extracted from 0.1 g leaf tissue infected by GFLV), lane 6: negative control (*E. coli* with pET26 empty vector used for expression steps), lane M: DNA molecular weight marker (1 kb).

## Data Availability

All data generated or analyzed during this study are included in the article and its additional files. Data sets from individual experiments can be obtained from the corresponding authors upon reasonable request.

## Abbreviations

BSA: Bovine serum albumin
CCMV: Cowpea chlorotic mottle virus
CE: Capillary electrophoresis
CP: Capsid protein
CPMV: Cowpea mosaic virus
DTT: Dithiothreitol
GFLV: Grapevine fanleaf virus
HPV: Human papillomavirus
IB: Inclusion body
IPTG: Isopropyl β-D-1-thiogalactopyranoside
ISEM: Immunostaining electron microscopy
LB: Luria–Bertani
PDB: Protein Data Bank
PEO: Poly (ethylene oxide)
ProDA: Protein Design Assistant
SEC: Size exclusion chromatography
TEM: Transmission electron microscopy
VLP: Virus-like particle
