# Supplementary figures and images for: Production and characterization of virus-like particles of grapevine fanleaf virus presenting L2 epitope of human papillomavirus minor capsid protein
Source: BMC Biotechnol. 2019 Nov 21;19:81. doi: 10.1186/s12896-019-0566-y (PMC6868843; doi:10.1186/s12896-019-0566-y)

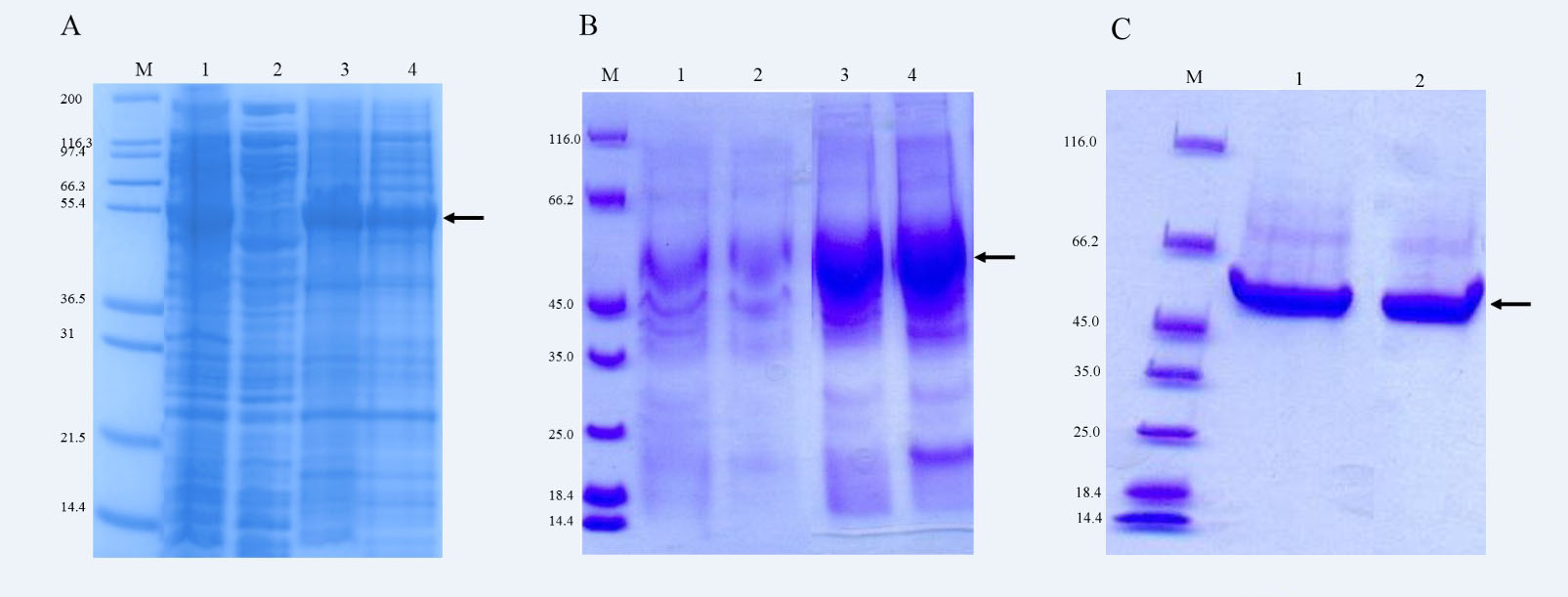

Supplement: Supplementary file 1 — Additional file 1: Figure S1. (A) SDS–PAGE analysis of the protein expression in E. coli. Lane M: unstained PageRuler® molecular weight markers, lane 1: Total fraction of GFLV-L2, lane 2: soluble fraction of GFLV-L2, lane 3: insoluble fraction of GFLV-L2, lane 4: insoluble fraction of GFLV CP. (B) SDS–PAGE analysis after application of the solubilisation protocol. Lane M: unstained PageRuler® molecular weight markers, lane 1: pellet fraction of GFLV CP, lane 2: pellet fraction of GFLV L2, lane 3: supernatant fraction of GFLV CP, lane 4: supernatant fraction of GFLV L2. (C) SDS–PAGE analysis after sucrose cushion ultracentrifugation. Lane M: unstained PageRuler® molecular weight markers, lane 1: GFLV CP VLPs, lane 2: GFLV L2 VLPs. The proteins of interest are indicated by an arrow. Gels were stained with coomassie blue. [file 12896_2019_566_MOESM1_ESM.jpg]

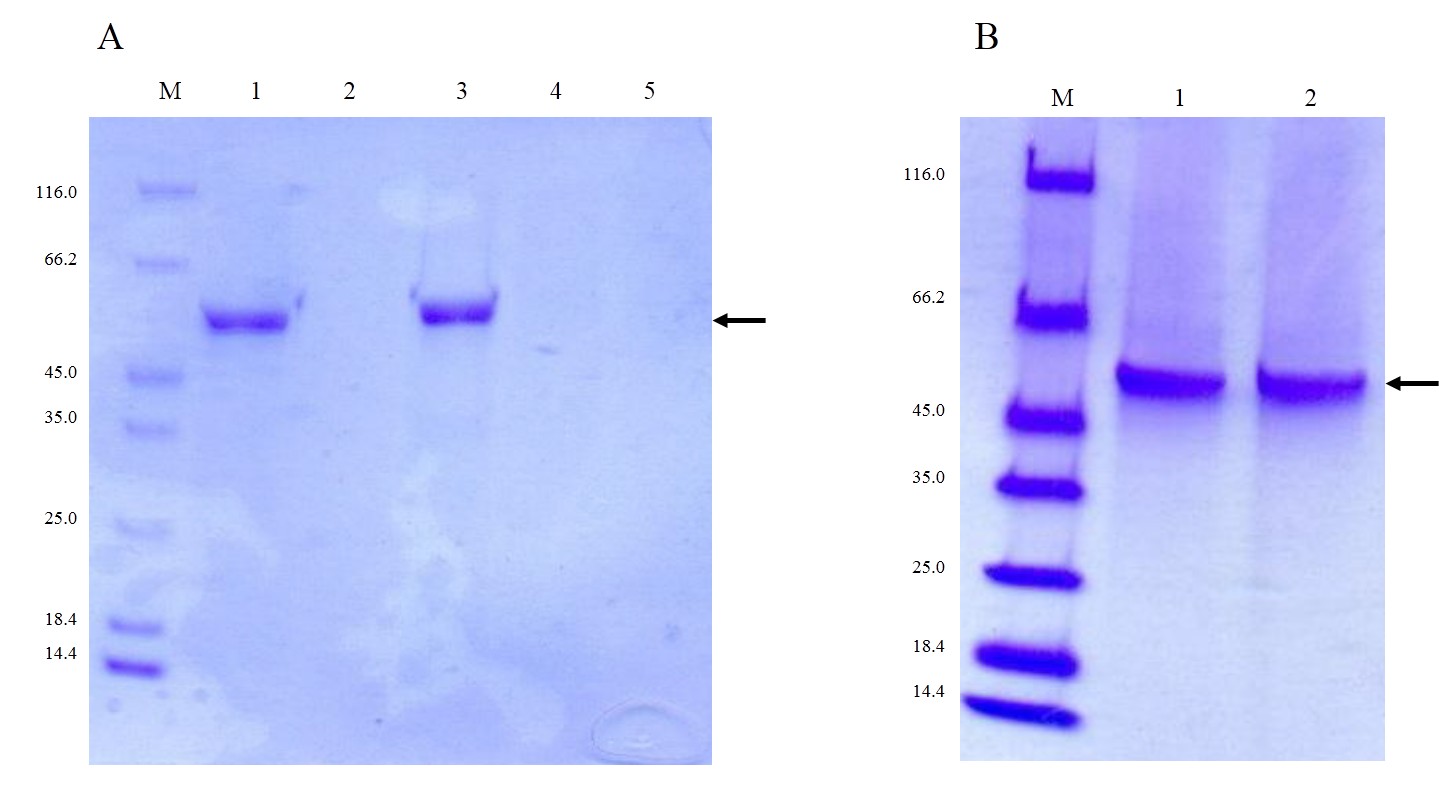

Supplement: Supplementary file 2 — Additional file 2: Figure S2. (A) SDS-PAGE analysis of the GFLV VLP samples expressed in P. pastoris. Lane M: unstained PageRuler® molecular weight markers, lane 1: clone A1 of GFLV CP, lane 2: clone A2 of GFLV CP, lane 3: clone A3 of GFLV L2, lane 4: clone A4 of GFLV L2, lane 5: negative control (P. pastoris with pPICZα empty vector). (B) SDS–PAGE analysis of the GFLV VLP samples expressed in P. pastoris after sucrose cushion ultracentrifugation. Lane M: unstained PageRuler® molecular weight markers, lane 1: clone A1 of GFLV CP, lane 2: clone A3 of GFLV L2. Gel was stained with coomassie blue. [file 12896_2019_566_MOESM2_ESM.jpg]

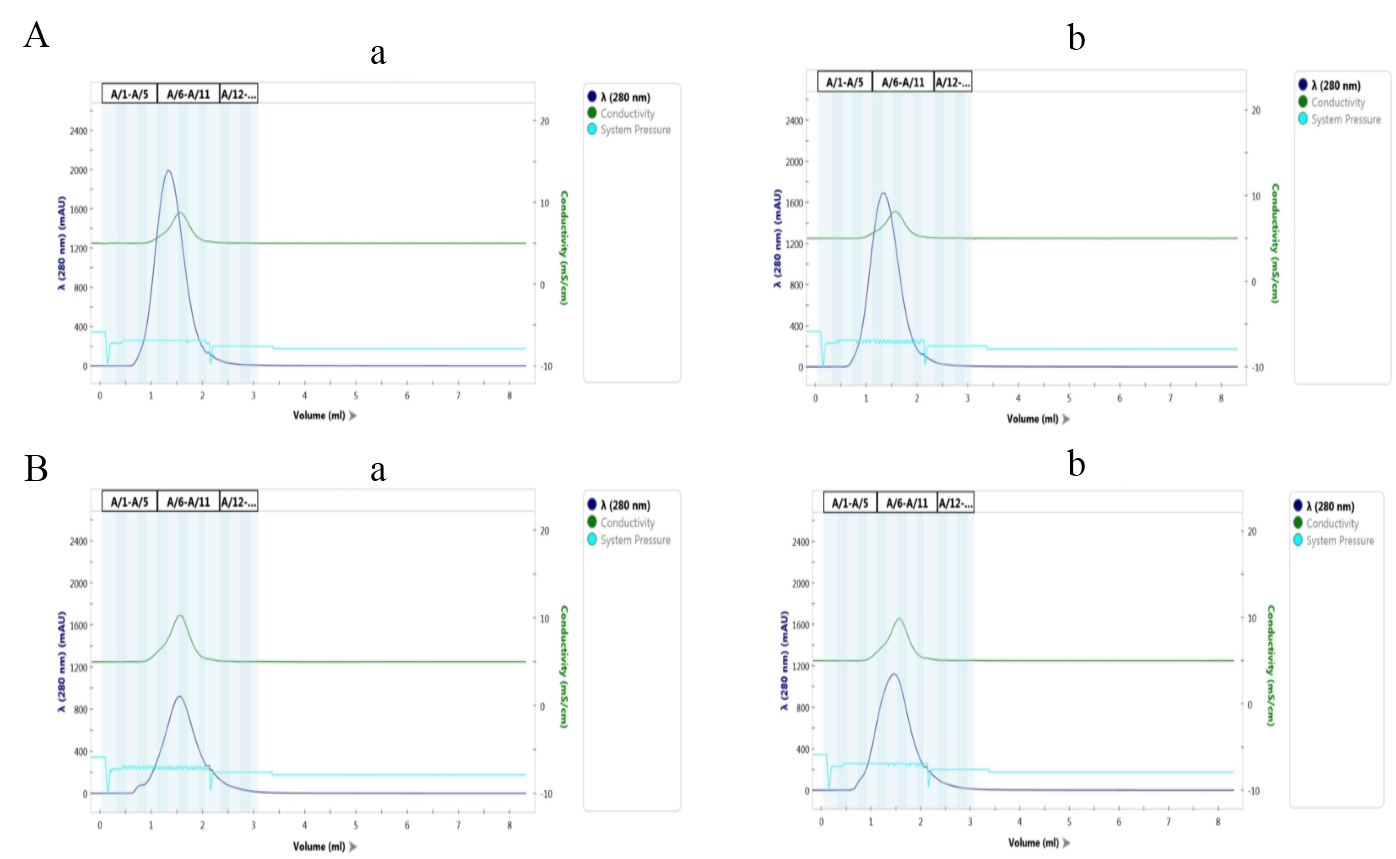

Supplement: Supplementary file 3 — Additional file 3: Figure S3. Size exclusion chromatography (SEC) analysis of GFLV VLPs using a Sephacryl 300 column (absorbance at 280 nm). (A) SEC analysis of the VLPs produced in E. coli, a GFLV CP VLPs, b GFLV L2 VLPs. (B) SEC analysis of the VLPs produced in P. pastoris, a GFLV CP VLPs, b GFLV L2 VLPs. [file 12896_2019_566_MOESM3_ESM.tif]

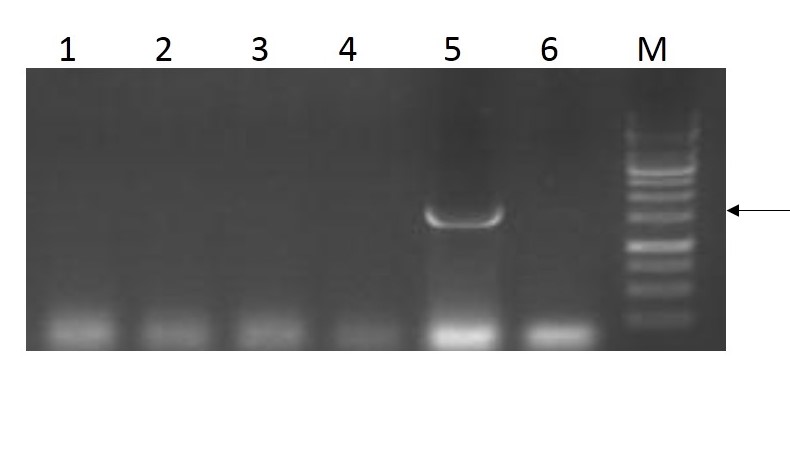

Supplement: Supplementary file 4 — Additional file 4: Figure S4. Analysis of RT-PCR products of GFLV CP and GFLV L2 mRNA by agarose gel electrophoresis. Lanes 1–2: nucleic acids extracted from 200 μL GFLV CP and GFLV L2 VLPs samples from E. coli, lanes 3–4: nucleic acids extracted from 200 μL GFLV CP and GFLV L2 VLPs samples from P. pastoris. Lane 5: positive control (i.e. nucleic acids extracted from 0.1 g leaf tissue infected by GFLV), lane 6: negative control (E. coli with pET26 empty vector used for expression steps), lane M: DNA molecular weight marker (1 kb). [file 12896_2019_566_MOESM4_ESM.jpg]
